# Eosinophilic Esophagitis in Saudi Children: Symptoms, Histology and Endoscopy Results

**DOI:** 10.4103/1319-3767.77242

**Published:** 2011

**Authors:** Mohammed Y. Hasosah, Ghassan A. Sukkar, Ashraf F. Alsahafi, Adel O. Thabit, Mohammed E. Fakeeh, Daifulah M. Al-Zahrani, Mohamed B. Satti

**Affiliations:** Department of Pediatric Gastroenterology, King Abdul-Aziz Medical City, National Guard Hospital, Jeddah, Saudi Arabia; 1 Department of Pediatric Immunology, King Abdul-Aziz Medical City, National Guard Hospital, Jeddah, Saudi Arabia; 2 Department of Pathology, King Abdul-Aziz Medical City, National Guard Hospital, Jeddah, Saudi Arabia

**Keywords:** Eosinophilic esophagitis, esophagogastroduodenoscopy, peripheral eosinophilia

## Abstract

**Background/Aim:**

Eosinophilic esophagitis (EE) is a clinicopathologic entity characterized by esophageal symptoms in association with a dense eosinophilic infiltrate currently defined as >15 eosinophils per high power field in the appropriate clinical context. This is the first pediatric study in Saudi Arabia to give the experience with EE and examine its symptom, histology and endoscopy results.

**Materials and Methods:**

Retrospective chart review of all patients diagnosed with EE at National Guard Hospital, Jeddah Between 2007 and 2009. The authors identified EE on histologic criteria (≥15 eosinophils per high-power field) together with their clinical context. The authors reviewed medical records for details of clinical presentation, laboratory data, radiologic, endoscopic, and histologic findings, and the results of treatment.

**Results:**

We identified 15 patients in our database in the last three years. 100% of the patients were males. The median age at presentation was 10 years (range, 3-17 years). The commonly reported symptoms were failure to thrive (86%), epigastric abdominal pain (53%), poor eating (40%), dysphagia with solid food (26%), food impaction (13%), and vomiting (20%). Asthma was reported in 46% and allergic rhinitis in 40%. Peripheral eosinophilia (>0.7 × 10/l) was found in 66%. High serum IgE Level (>60 IU/ml) was found in 60%. Upper endoscopic analysis revealed esophageal trachealization in 46%, esophageal erythema in 46%, white specks on the esophageal mucosa in 33%, esophageal narrowing in 13%, and normal endoscopy in 13%. The mean eosinophils per high-power field was 30.4 (range, 20–71). Histologic characteristics included degranulated eosinophils (86%), basal cell hyperplasia (93%) and eosinophils clusters (micro-abscess) in 73%. The treatment of EE revealed that they used swallowed corticosteroid in 50%, proton pump inhibitors in 66%, elemental diet/ food elimination in 13% and systemic corticosteroid in 13%.

**Conclusions:**

Failure to thrive and abdominal pain in a male, atopic school-aged child was the most common feature of EE. Peripheral eosinophilia, high serum IgE and endoscopic esophageal erythema and trachealization should significantly raise the clinical index of suspicion for the diagnosis of EE.

Eosinophilic esophagitis (EE) is a clinicopathologic entity characterized by esophageal symptoms in association with a dense eosinophilic infiltrate currently defined as >15 eosinophils per high power field (HPF) in the appropriate clinical context.[1] The International Gastrointestinal Eosinophil Researchers (TIGERS) undertook a systematic review of the published literature and finally created the following definition: EE is a clinicopathologic condition characterized by esophageal symptoms and a dense esophageal eosinophilia, both of which persist despite prolonged treatment with proton pump inhibitors (PPIs), whereas eosinophilic inflammation is absent in the other sections of the digestive tract.[1]

EE is increasingly recognized in both children and adults, and multiple studies document the rising prevalence of this disorder.[2] Pediatric patients with EE typically manifest vomiting, regurgitation, epigastric and chest pain, and water brash. Older children and adults may also experience heartburn and dysphagia. Although the symptoms are similar to those seen in gastroesophageal reflux disease (GERD), patients experience mild or no response to acid suppression and other forms of antireflux therapy.[3] Typically, the symptoms and histopathology of EE are unresponsive to proton pump inhibitor treatment. They are responsive to antiallergic treatments including dietary restriction and corticosteroids.[4]

To the best of our knowledge, no Saudi studies regarding EE in adult or children have been reported yet. This is the first pediatric study in Saudi Arabia to give the experience with EE and examine its symptom, histology and endoscopy results.

## PATIENTS AND METHODS

### Patient selection

Retrospective review and analysis of 351 patients who underwent esophagogastroduodenoscopy (EGD) with mucosal biopsies between January 1, 2007 and December, 2009 at King Abdul-Aziz Medical City, National Guard Hospital, Jeddah, Saudi Arabia. The patients with EE were identified by electronic and manual search.

### Methods

The diagnosis of EE was made in patients who had at least one of the following symptoms: failure to thrive, vomiting, regurgitation, abdominal pain, food impaction, or dysphagia unresponsive to a two-month therapeutic trial of proton pump inhibitors (PPIs). To confirm the diagnosis, patients underwent esophagogastroduodenoscopy (EGD) with biopsies obtained from the proximal, mid, and distal esophagus, stomach, and duodenum. Additionally, patients also underwent colonoscopies if clinically warranted. The biopsies were evaluated under ×40 HPF and reviewed by a board certified pathologist. Biopsies obtained from 1 to 3 esophageal levels (distal, mid, and/or proximal) were fixed in 10% formalin, processed routinely, and stained with hematoxylin and eosin. The number of esophageal specimens per endoscopic procedure ranged from 1 to 4 with 1 to 2 biopsies obtained per level. EE was diagnosed if patients had greater than 15 eosinophils in the most densely involved HPF in the esophagus with normal stomach and duodenal biopsies together with their clinical context.[1]

Patients who fulfilled the following criteria were enrolled in the study: 1-Age less than 14 years. 2-Histological criteria of EE: >15 eosinophils per HPF. 3- Positive history of food allergy. 4-Patients who did not respond to treatment of gastroesophageal reflux disease (GERD).

Exclusion criteria were the following: 1- Patients who did not meet the histologic criteria for EE. 2-Patients with eosinophilic gastroenteritis, viral or fungal esophagitis. 3-History of drug allergy 4-History of concomitant illness (eg, inflammatory bowel disease, celiac disease).

Patient demographics, clinical presentation, laboratory data, radiologic findings, endoscopic findings, histological findings and the results of treatment were retrieved from the patients’ medical records. This study was approved by the Research Committee of National Guard Health Affairs, Western Region.

## RESULTS

We identified 17 patients in our database in the last three years. Two patients were excluded because they did not fulfill the diagnostic criteria of EE. The net total numbers of EE were 15 patients.

### Clinical characteristics of EE

The median age at presentation was 10 years (range, 3-17 years). 100% of the patients were males. The clinical characteristics of EE are shown in Table 1. The commonly reported symptoms of EE were failure to thrive in thirteen patients (86%), epigastric pain in eight of fifteen patients (53%), poor eating in six patients (40%), dysphasia with solid food in four patients (26%), vomiting in three patients (20%) and food impaction in two patients (13%).

**Table 1:** The clinical characteristics of EE

**Presenting feature**	**No. of patients (%)**
Failure to thrive	13 (86)
Epigastric abdominal pain	8 (53)
Poor eating	6 (40)
Dysphagia	4 (26)
Vomiting	3 (20)
Food bolus impaction	2 (13)
Bronchial asthma	7 (46)
Allergic rhinitis	6 (40)
Peripheral eosinophilia	10 (66)
High serum IgE	9 (60)
Positive aeroallergen RAST	9 (60)

EE: Eosinophilic esophagitis, RAST: radioallergosorbent testing

Bronchial asthma was reported in seven patients (46%) and allergic rhinitis in six patients (40%). Peripheral eosinophilia (>0.7 × 10/l) was found in ten patients (66%). High serum IgE level (>60 iu/ml) was found in nine patients (60%). Specific serum IgE antibodies to casein, lactoglobulin, nuts, soy, peanuts, eggs, and wheat were not done.

Nine patients with EE had radioallergosorbent testing (RAST) positive for at least one food (60%). Food allergy was reported in six patients, while other patients were waiting for skin prick test (data is not shown in Table 1).

An upper gastrointestinal series was performed in three patients only. In two, narrowing of the esophageal lumen was observed. Esophageal pH probe monitoring, performed in four patients, showed normal finding.

### Endoscopic characteristics of EE

**Figure 1:**
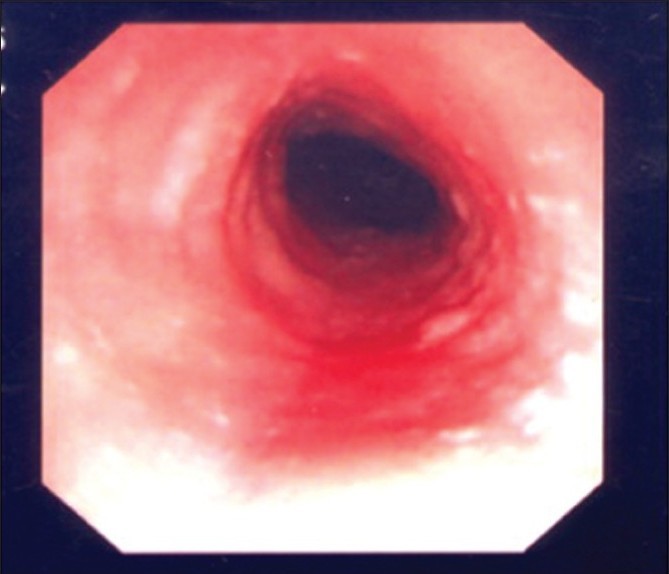
Endoscopic findings of EE showing esophageal erythema and trachealization

Endoscopic characteristics are shown in Table 2. Thirteen children with EE had esophageal abnormalities. Esophageal erythema and trachealization were the most common endoscopic findings in EE patients [Figure 1].

Upper endoscopic analysis revealed esophageal trachealization in seven patients (46%), esophageal erythema in seven patients (46%), white specks on the esophageal mucosa in five patients (33%), esophageal narrowing in two patients (13%), and normal endoscopy in two patients (13%). Patients with white speckles or plaques were often presumed to have candida esophagitis, but this was subsequently excluded by negative microscopic and microbiologic testing.

**Table 2:** Endoscopic, histologic and treatment of EE

**Feature**	**No. of patients (%)**
Esophageal trachealization	7 (46)
Esophageal erythema	7 (46)
Esophageal white specks	5 (33)
Esophageal narrowing	2 (13)
Normal endoscopy	2 (13)
Median eosinophils/HPF	30.4
Degranulated eosinophils	12 (86)
Basal cell hyperplasia	14 (93)
Eosinophils clusters (micro-abscess)	11 (73)
Oral corticosteroids	2 (13)
Swallowed inhaled fluticasone	8 (53)
Proton pump inhibitor	10 (66)
Elemental diet	2 (13)
Elimination diet	2 (13)

EE: Eosinophilic esophagitis, HPF: High power field

### Histologic characteristics and treatment of EE

**Figure 2:**
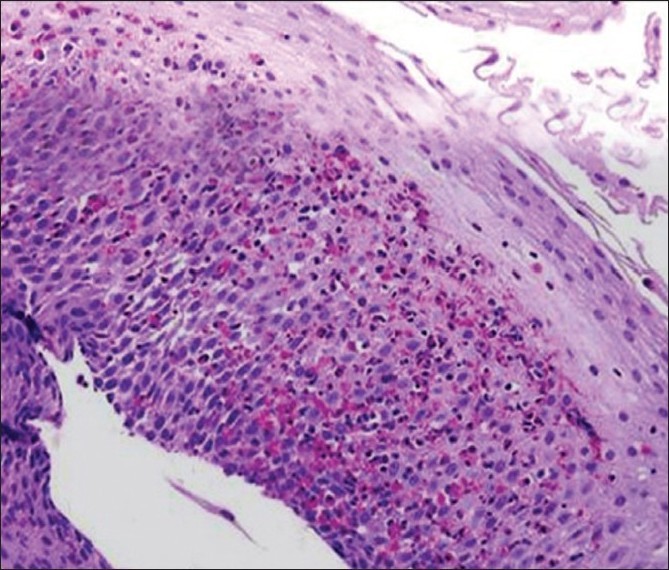
Histologic findings showing infiltration with eosinophils and eosinophil granules

Histologic characteristics are shown in Table 2. The mean number of eosinophils per HPF in EE patients was 30.4 (range, 20–71) [Figure 2]. Histological characteristics included degranulated eosinophils in 86%, basal cell hyperplasia in 93% and eosinophils clusters (micro-abscess) in 73%.

The treatment of EE revealed that eight patients used swallowed inhaled corticosteroid (50%), ten patients used proton pump inhibitors (66%), two patients used elemental diet/ food elimination (13%) and two patients used systemic corticosteroid (13%).

Only one patient had repeated endoscopy to assess the number of eosinophils and response to EE treatment while others were refused repeating endoscopy.

## DISCUSSION

This is the first case series study in Saudi Arabia reporting EE. In this report, we demonstrate the clinical, endoscopic, and histologic characteristics of patients with EE in pediatric population. However, only one case was reported by Al-Hussaini *et al*.,[5] which showed a child had esophageal trachealization as feature of EE.

EE is defined by infiltration of the esophagus with eosinophils without infiltration in other parts of the gastrointestinal tract.[1] As an emerging syndrome, the diagnostic criteria for EE are unsettled. Although there is an agreement that a patchy, panesophagitis with eosinophil levels greater than 20 per HPF is diagnostic for EE, some experts have suggested that levels between 5 and 20 are ambiguous as to the correct diagnosis.[6–9]

We chose to use a cut-off of 15 eosinophils per HPF at any esophageal level with the understanding that there may be children with EE who may not be included in the study, due to this definition. The American Gastroenterological Association (AGA) defined EE as having greater than 15 eosinophils /HPF in a single esophageal biopsy.

EE has been increasingly diagnosed in the recent years.[10] We continue to see an increase in the number of newly diagnosed patients with EE in our local population. The reasons for the increase in the number of newly diagnosed EE patients include increased EGDs with esophageal biopsies owing to the ease of endoscopic evaluation, or increase in patient population or increased recognition of the disorder.

The etiology of EE is not known. Increasing emphasis has been placed on the role of food allergy,[11] but EE may also be a subset of eosinophilic gastroenteritis, an autoimmune disorder. Although the etiology of the rise of EE remains unclear, it is probably similar to the increase seenin other atopic diseases such as asthma and atopic dermatitis.[12]

Our EE population is highly atopic, with 50% of the patients having other allergic diseases. This suggests that allergic disease in another organ system can affect the esophagus, similar to the link with asthma and allergic rhinitis (nose and lung).[13]

Previous studies also have found a male predominance, with a majority of the patients being atopic, ranging from 33% to 70% in different studies[14–16] which are similar to our study.

The symptoms of EE have been described as symptoms suggestive of gastroesophageal reflux, which do not respond to gastroesophageal reflux disease (GERD) medications. Other symptoms of EE include dysphagia in older children and failure to thrive in infants.[17,18]

Because most of our patients are younger than nine years, it is not surprising that failure to thrive/feeding issues and GERD-like symptoms are the most common presentations, given that this is the presentation seen in the youngest children.

Owing to similar clinical presentations of vomiting, pain, and dysphagia, the distinction of EE from more common non-EE diagnoses, such as GERD, can be difficult. This is why PH probe is not done in all patients.

As the frequency of esophageal erythema and trachealization is higher in our patients with EE, other findings of white specks on the esophageal mucosa and esophageal narrowing should also greatly increase the suspicion of EE.

Ultimately, the diagnosis of EE depends on the presence of elevated numbers of eosinophils at multiple esophageal levels. Our histologic results underscore the importance of obtaining multiple esophageal biopsy specimens at varying esophageal levels as a routine part of esophagoduodenoscopy. Only EE patients demonstrated esophageal desquamation, eosinophil clusters and these histologic features should be considered as pathognomonic for EE.

Studies on adults and children demonstrate the presence of fibrosis in EE patients and may point to another distinguishing feature of EE,[19,20] Our study cannot demonstrate lamina propria fibrosis.

Our study showed varied treatment of EE which include the use of topical steroids, proton pump inhibitor (PPI) monotherapy, and elemental diets which were similar to previous studies.[21–23]

Despite the limitation of this retrospective small analysis that cannot be controlled for clinical history and biopsy collection, these data clearly demonstrate that a significant proportion of atopic children who are referred for esophagoduodenoscopy with biopsy have a diagnosis of EE. Nevertheless, the data suggest that further studies, especially a prospective, randomized evaluation of EE outlined in this review, are warranted.

## CONCLUSION

We demonstrate that a male, atopic school-aged child who complains of poor growth and abdominal pain should significantly raise the clinical index of suspicion for the diagnosis of EE. If peripheral eosinophilia and high serum IgE are present, the diagnosis of EE becomes increasingly likely. Lastly, if these clinical complaints are combined with endoscopic findings of esophageal erythema, trachealization, or strictures, the histologic diagnosis will almost certainly be EE. Prompt referral of atopic patients with upper GI symptoms, especially school-aged boys, to pediatric gastroenterology and allergy services will hopefully lead to appropriate diagnosis and therapy in children with EE.
